# TMED3 promotes hepatocellular carcinoma progression via IL-11/STAT3 signaling

**DOI:** 10.1038/srep37070

**Published:** 2016-11-30

**Authors:** Hao Zheng, Yuan Yang, Jun Han, Wei-hua Jiang, Cheng Chen, Meng-chao Wang, Rong Gao, Shuai Li, Tao Tian, Jian Wang, Li-jun Ma, Hao Ren, Wei-ping Zhou

**Affiliations:** 1 The Third Department of Hepatic Surgery, Eastern Hepatobiliary Surgery Hospital, Second Military Medical University, 225 Changhai Road, Shanghai 200438, China; 2 Department of Health Statistics, Second Military Medical University, 800 Xiangyin Road, Shanghai 200433, China; 3 Department of Microbiology, Shanghai Key Laboratory of Medical Biodefense, Second Military Medical University, 800 Xiangyin Road, Shanghai 200433, China; 4 Department of Oncology, Shanghai Tongren Hospital, Shanghai Jiaotong University, 1111 Xianxia Road, Shanghai 200336, China; 5 Department of Medical Oncology, Jinling Hospital, 305 Zhongshan East Road, Nanjing, Jiangsu 210000, China; 6 Department of Computer Science, Rensselaer Polytechnic Institute, 110 8th Street, Troy, NY, 12180, United States

## Abstract

Transmembrane p24 trafficking protein 3(TMED3) is a metastatic suppressor in colon cancer, but its function in the progression of hepatocellular carcinoma (HCC) is unknown. Here, we report that TMED3 was up-regulated in HCC and portal vein tumor thrombus. TMED3 up-regulation in HCC was significantly correlated with aggressive characteristics and predicted poor prognosis in HCC patients. TMED3 overexpression in HCC cell lines promoted cell migration and invasion. In contrast, TMED3 knockdown suppressed HCC metastasis both *in vitro* and *in vivo*. Gene microarray analysis revealed decreased IL-11 expression in TMED3-knockdown cells. We propose that TMED3 promotes HCC metastasis through IL-11/STAT3 signaling. Taken together, these findings demonstrate that TMED3 promotes HCC metastasis and is a potential prognostic biomarker in HCC.

Hepatocellular carcinoma (HCC) is one of the most lethal malignancies^1^,^2^,^3^. With improvements in surgical techniques and perioperative management, the five-year survival rates for HCC after curative therapy have somewhat increased^2^,^4^,^5^. However, the rates of recurrence and intrahepatic or extrahepatic metastasis after surgery remain high^6^,^7^,^8^, thus limiting the prognosis for HCC patients^9^,^10^. Therefore, a comprehensive understanding of the molecular pathogenesis of HCC recurrence and metastasis is imperative.

The transmembrane emp24 domain-containing protein (TMED)/p24 family is involved in the vesicular trafficking of proteins and innate immune signaling. The ten mammalian family members, which are conserved across species, are separated into four subfamilies (α, β, δ, γ)^11^. TMED proteins primarily exist as monomers, dimers, oligomers and hetero-oligomers in eukaryotes^12^,^13^,^14^. These proteins contain a GOLD (Golgi dynamics) domain, which is a β-strand-rich domain found in several proteins that are involved in Golgi dynamics and intracellular protein trafficking^13^,^15^. Many TMED proteins have been investigated and reported on in detail^16^,^17^. TMED7, which negatively regulates TLR4 signaling, was identified as a specific inhibitor of the MyD88-independent TLR4 signaling pathway through its ability to facilitate the disruption of the TRIF/TRAM complex by TAG^18^,^19^,^20^. However, the clinical significance of TMED3 and its role in HCC pathogenesis remain unknown.

In the present study, we demonstrate that TMED3 was up-regulated in HCC tissues and was expressed at even higher levels in portal vein tumor thrombus (PVTT). TMED3 up-regulation was associated with poor clinical outcome in HCC patients. TMED3 down-regulation dramatically attenuated HCC migration both *in vitro* and *in vivo*. A gene microarray analysis of a TMED3-knockdown cell line revealed low IL-11 expression. These findings suggest that TMED3 plays an important role in HCC progression through IL-11/STAT3 signaling and may be useful for future HCC therapy.

## Results

### TMED3 expression is up-regulated in HCC and PVTT

**Figure 1:**
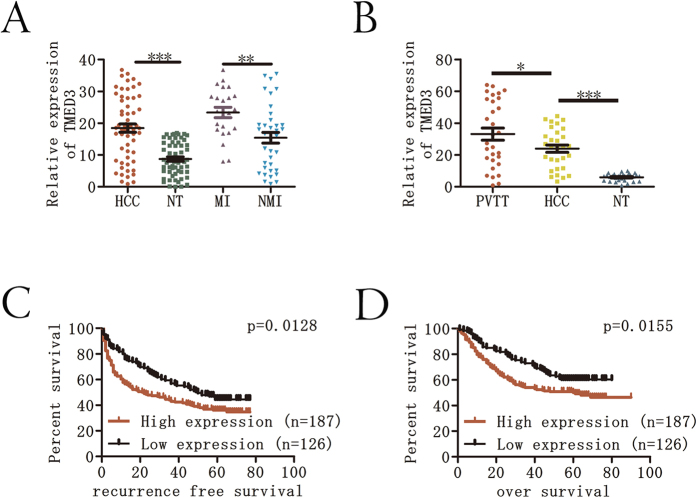
TMED3 expression is up-regulated in HCC and in cases of PVTT. (**A**) Relative TMED3 mRNA expression levels in 60 cases of HCC, including 25 MI and 35 NMI, were analyzed by RT-PCR and normalized to β-actin. (**B**) Relative TMED3 mRNA expression levels in 30 cases of HCC with PVTT were analyzed by RT-PCR and normalized to β-actin. (**C**) The high TMED3 expression group had a shorter RFS than the low TMED3 expression group. (**D**) The high TMED3 expression group had a shorter OS than the low TMED3 expression group. *p < 0.05, **p < 0.01, ***p < 0.001.

To clarify the underlying role of TMED3 in HCC progression, we performed qRT-PCR analysis of 60 HCC specimens and paired non-tumor (NT) samples. TMED3 mRNA levels were significantly up-regulated in most HCC tissues (Fig. 1A). We next examined TMED3 protein levels by immunohistochemistry (IHC) on a tissue microarray containing 313 paired HCC tissues. Immunostaining revealed that 63.9% (200/313) of the HCC tissues showed higher TMED3 protein levels than their corresponding adjacent noncancerous tissues (Fig. S1B and C). To further investigate the role of TMED3 in HCC metastasis, we examined TMED3 expression in primary metastatic or non-metastatic HCC. TMED3 expression was higher in metastasis-inclined (MI) HCC than in non-metastasis-inclined (NMI) HCC (Fig. 1A). Furthermore, we examined TMED3 expression in 30 matched PVTT, primary tumor and NT tissues. Intriguingly, TMED3 mRNA levels were significantly higher in PVTT than in primary tumors or NT tissues (Fig. 1B), suggesting that TMED3 may play a role in HCC metastasis. To investigate the clinical significance of TMED3 in HCC, the cohort of 313 HCC patients was divided into two groups according to the tumor IHC score: a high TMED3 expression group (tumor score > 2, n = 187) and a low TMED3 expression group (tumor score ≤ 2, n = 126). High TMED3 expression was associated with positive alpha-fetoprotein (AFP; P = 0.020), larger tumor size (≥5 cm; P = 0.045) and vascular invasion (P < 0.001) (Table 1). Kaplan–Meier analysis revealed that patients in the high TMED3 expression group exhibited worse relapse-free survival (RFS) and overall survival (OS) than patients in the low expression group (P = 0.0128 and P = 0.0155, respectively) (Fig. 1C and D). A univariate analysis indicated that among the clinicopathological characteristics, TMED3 expression level, tumor size, vascular invasion, and positivity for AFP, HBsAg, or HBeAg were correlated with RFS, and TMED3 expression level, tumor size, tumor number, AFP positivity, vascular invasion, presence of HBsAg, and presence of HBeAg were correlated with OS (Table 2). Furthermore, multivariate Cox regression analysis indicated that TMED3 expression level, tumor size, presence of HBeAg, and presence of HBsAg were independent risk factors for RFS, and TMED3 expression level, tumor size, tumor number, and presence of HBsAg were independent risk factors for OS in HCC patients (Table 3). These results indicated that TMED3 could be used as a prognostic biomarker for HCC.

### TMED3 promotes HCC cell metastasis *in vitro*

**Figure 2:**
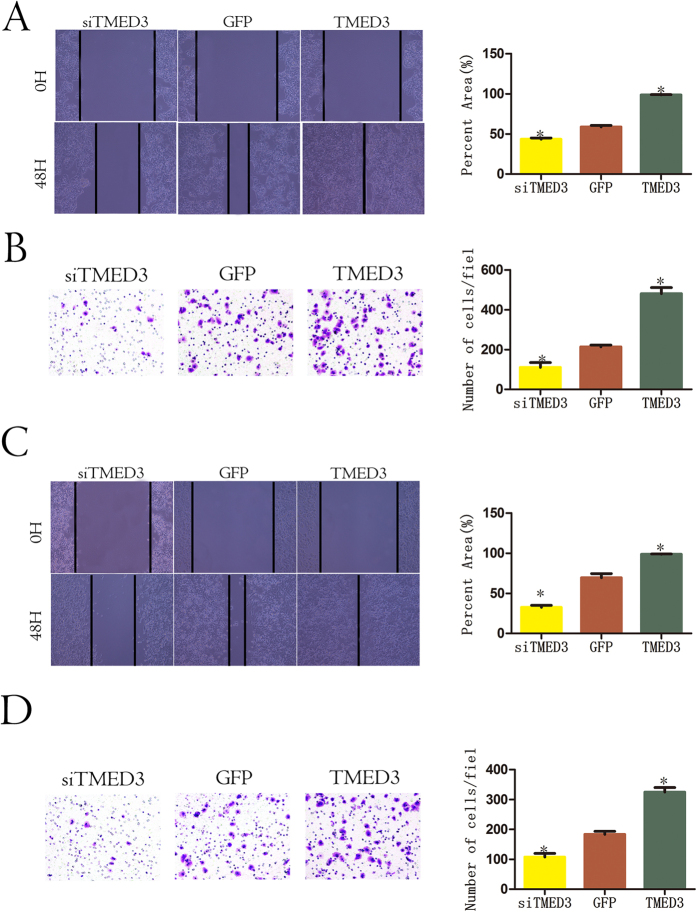
TMED3 promotes HCC metastasis *in vitro*. Wound healing and transwell migration assays showed that TMED3 knockdown inhibits migratory properties and that TMED3 overexpression promotes migration in the HCC-derived cell lines HepG2 (**A**,**B**) and Huh7 (**C**,**D**). Representative results and a statistical analysis are shown. (**A–D**) n = 3, mean ± SD. The Student’s t test, *p < 0.05 versus GFP control.

To explore the biological functions of TMED3 in HCC *in vitro*, we first detected TMED3 expression in a normal human liver cell line (HL7702) and in HCC cell lines with varying metastatic potential (HCCLM3, MHCC97H, MHCC97L, SMMC-7721, Huh7, HepG2, Hep3B). TMED3 expression levels were higher in the highly metastatic HCC cell lines (HCCLM3, MHCC97H) than in the less metastatic cell lines (SMMC-7721, MHCC97L); the normal liver cell line had the lowest TMED3 expression (Fig. S2A). Two HCC cell lines (HepG2, Huh7) with median TMED3 expression were selected to establish TMED3-knockdown or TMED3-overexpressing cells. TMED3 expression was validated by qRT-PCR (Fig. S2B and C). A wound healing migration assay showed that TMED3 knockdown inhibited cell migration, whereas TMED3 overexpression enhanced cell mobility (Fig. 2A and C). A transwell invasion assay revealed that TMED3-knockdown cells displayed impaired migration, whereas cells overexpressing TMED3 exhibited increased invasiveness (Fig. 2B and D). These results indicated that TMED3 promotes HCC metastasis *in vitro*.

### TMED3 promotes HCC metastasis *in vivo*

**Figure 3:**
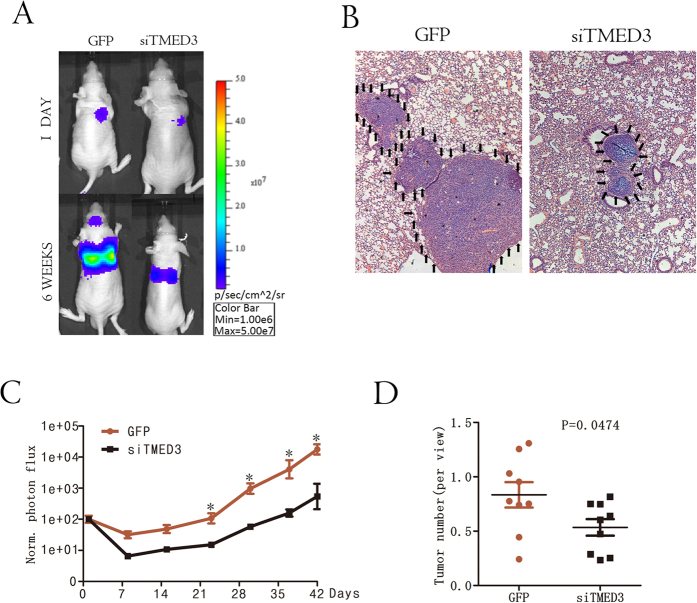
TMED3 promotes HCC metastasis *in vivo*. (**A**) Lung metastases that developed after injecting HepG2 cell lines in the lateral tail vein were imaged using the IVIS Imaging System. Representative luciferase signals captured in each group (n = 9) at initial injection and after 42 days are shown. The statistical analysis is shown in (**C**), n = 9, mean ± SD, the Student’s t test, *p < 0.05 versus GFP control. (**B**) Representative imaged of H&E-stained lung metastatic loci from each group in (**A**). The statistical analysis is shown in (**D**).

To verify the function of TMED3 *in vivo*, we injected luciferase-expressing HepG2-siTMED3 and HepG2-GFP cells into the lateral tail vein of mice to establish a lung metastasis model. The process of lung metastasis was dynamically monitored every week using an *in vivo* imaging system. Bioluminescent imaging revealed that TMED3 knockdown suppressed HCC lung metastasis (Fig. 3A and C). After 42 days, the lungs were dissected and stained with hematoxylin and eosin (H&E). Fewer micrometastases were microscopically detected in the lungs of mice in the TMED3-knockdown group (Fig. 3B and D). Additionally, we injected HepG2-siTMED3 and HepG2-GFP cells subcutaneously into nude mice. Weekly tumor volume measurements showed that TMED3 had no effect on cell growth (Fig. S3A and B). These data demonstrated that TMED3 may promote the metastasis of hepatoma cells *in vivo*.

### TMED3 promotes cell migration by increasing IL-11 expression

**Figure 4:**
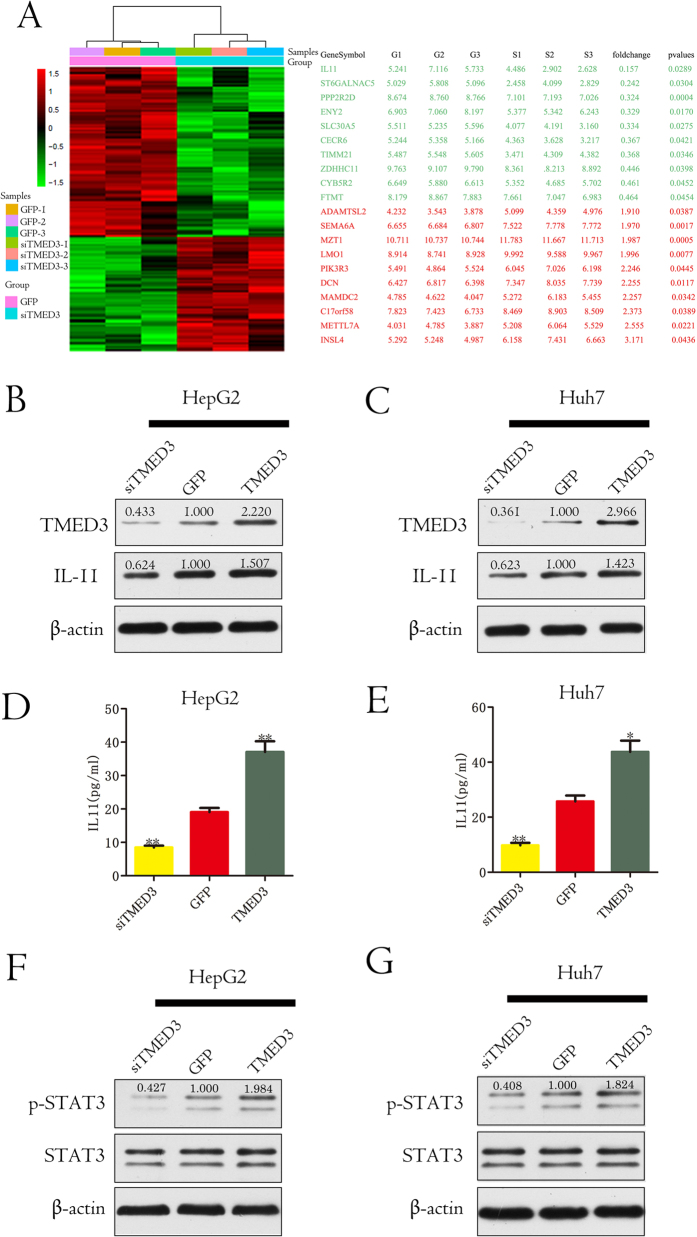
TMED3 promotes cell migration by increasing IL-11 expression. (**A**) Differentially expressed genes in HepG2-siTMED3 cells compared with control cells. The top 10 up- and down-regulated genes are listed on the right. (**B**,**C**) Relative IL-11 expression in cells with stable TMED3 knockdown or overexpression and paired controls. IL-11 expression was analyzed by western blot and normalized to β-actin. (**D**,**E**) The IL-11 concentration in culture medium from cells with stable TMED3 knockdown or overexpression and paired controls was determined by ELISA. n = 3, mean ± SD, the Student’s t test, *p < 0.05 versus GFP control, **p < 0.01 versus GFP control. (**F**,**G**) Relative P-STAT3 and STAT3 expression levels in cells with stable TMED3 knockdown or overexpression and paired controls were analyzed by western blot and normalized to β-actin. (**B–G**) n = 3.

To explore the molecular mechanism of TMED3 in HCC cell metastasis, gene microarray analysis was utilized to compare the expression profiles of TMED3-knockdown and control HCC cells. A total of 64 genes were significantly up-regulated, and 95 genes were down-regulated (>1.5-fold, siTMED3/GFP). Interleukin 11(IL-11), which has been reported to regulate metastasis and cell adherence^21^,^22^, was the most robustly down-regulated gene in HepG2-siTMED3 cells (Fig. 4A). Western blotting validated IL-11 up-regulation in cells overexpressing TMED3 and down-regulation in TMED3-knock down cells (Fig. 4B and C). To examine whether TMED3 increases IL-11 secretion, we measured IL-11 levels in the supernatant from different cell clones. TMED3 overexpression increased IL-11 levels in the supernatant, and TMED3 knockdown reduced IL-11 levels in the supernatant (Fig. 4D and E). These results indicated that TMED3 up-regulates IL-11 expression, leading to increased IL-11 secretion. As Signal transducer and activator of transcription 3(STAT3) is the main signaling molecule downstream of IL-11, we also measured STAT3 phosphorylation levels. TMED3 overexpression led to increased STAT3 phosphorylation, and TMED3 knockdown decreased STAT3 activation (Fig. 4F and G). Importantly, a strong positive correlation between TMED3 and STAT3 phosphorylation levels was observed in clinical samples (Fig. S4). This finding suggested that TMED3 may promote metastasis at least partially through IL-11/STAT3 signaling.

## Discussion

Despite the available advanced surgical and medical treatments^1^,^2^, HCC recurrence and metastasis rates remain high due to the long-term interactions between environmental and genetic factors^23^, and the prognosis of HCC patients is still unsatisfactory^24^,^25^,^26^. Although multiple tumor suppressor genes and oncogenes involved in HCC have been identified^27^,^28^,^29^,^30^,^31^, our knowledge of the underlying cellular and molecular pathways in HCC progression remains limited. The molecular markers that effectively define the risk of HCC recurrence need to be identified and characterized to predict patient outcome and determine optimal medical management strategies.

In a previous study, TMED3 was found to be a metastatic suppressor in colon cancer^32^, but the function of TMED3 in HCC progression was unknown. In this study, we explored the clinical significance and role of TMED3 in HCC progression by measuring TMED3 protein levels in a large cohort of 313 patients. TMED3 up-regulation correlated with a high AFP level (P = 0.020), tumor size (P = 0.045) and vascular invasion (P < 0.001). Kaplan–Meier analysis revealed that patients with higher TMED3 expression had higher recurrence rates (P = 0.0128) and shorter survival times after curative resection (P = 0.0155), indicating that TMED3 expression is associated with HCC progression and may serve as an independent prognostic marker of HCC patient survival. To further investigate the effects of TMED3 in HCC cells, we performed gain- and loss-of-function experiments. Our data showed that TMED3 down-regulation suppressed HCC migration and invasion *in vitro* and lung metastasis *in vivo*. Thus, TMED3 can functionally promote HCC cell metastasis.

IL-11 belongs to the IL-6 family^33^,^34^, and similar to most cytokines in this family, IL-11 is produced by various cells in response to inflammatory stimulators. IL-11 exhibits a wide variety of biological effects in the hematopoietic and immune systems^35^,^36^,^37^. It has been reported that IL-11 promotes the development of gastric, breast, and colorectal cancer and contributes to the bone metastasis of HCC^38^,^39^,^40^. STAT proteins are potent, conserved transcription factors. Seven STAT proteins have been identified as latent cytoplasmic transcription factors activated by tyrosine phosphorylation in response to cytokine and growth factor stimulation^41^,^42^. STAT signaling plays an important role in the transfer of extracellular signals into the nucleus, resulting in transcriptional regulation, and is essential in the uncontrolled growth of cancer cells, angiogenesis and metastasis^43^,^44^. In our gene microarray analysis, we observed decreased IL-11 expression in TMED3-knockdown cells. Moreover, STAT3 activation correlated with TMED3 expression. These results suggested that TMED3 may promote HCC metastasis through IL-11/STAT3 signaling. However, the relationship between TMED3 and IL-11 remains unclear and requires further investigation.

In conclusion, our study determined that TMED3 is a valuable prognostic biomarker for HCC and revealed the role and potential mechanism of action of TMED3 in HCC metastasis. A complete understanding of TMED3 function and mechanism of action will reveal novel strategies for HCC treatment.

## Materials and Methods

### Ethics statement

All the clinical specimens were obtained with informed consent and approved by the the Clinical Research Ethics Committee of Eastern Hepatobiliary Surgery Hospital, Informed consent was obtained from all patients involved in this study. Animal experiments were reviewed and approved by the Institutional Animal Care and Use Committee of the Second Military Medical University. All the experiments were performed in accordance with the approved guidelines of the Institutional Research Ethics Committee of the Second Military Medical University University.

### Patients and specimens

This study included 60 HCC tissues and paired NT tissues (including 25 MI and 35 NMI) which were obtained from the Eastern Hepatobiliary Surgery Hospital for qRT-PCR analysis and these tissues were aimed to investigate the mRNA level of TMED3 and the clinicopathologic characteristic was shown in supplement Table 1. The MI group was composed of patients with solitary HCC that was accompanied by either portal vein metastasis or venous metastases at follow-up. The NMI group was restricted to only those patients with solitary HCC and no recurrence at follow-up. For further confirmation that TMED3 may be associated with tumor metastasis, other samples from 30 patients with HCC and PVTT were used to evaluate TMED3 expression levels in paired HCC/NT/PVTT samples by qRT-PCR. The clinicopathologic characteristic was shown in supplement Table 2. To study the correlation of TMED3 with clinical characteristics and prognosis, a large cohort of 313 patients with HCC (randomly collected from January 2006 to September 2010) who received high-quality follow-up was used to examine TMED3 protein levels by tissue microarray (TMA) and IHC and to ascertain the clinical significance of TMED3. The clinicopathologic characteristics were shown in Table 1. All HCC specimens were obtained immediately after hepatectomy. Tissues were then fixed in 10% buffered formalin and embedded in paraffin. The fresh specimens used in this study were snap-frozen from tissues prior to formalin fixation, transferred to liquid nitrogen, and stored at −80 °C.

### Tissue microarray construction and immunohistochemical analysis

Briefly, all samples from patients in the HCC cohort were reviewed histologically after H&E staining. Representative areas distant from necrotic and hemorrhagic tissue were marked on the paraffin blocks. Two 1.0-mm cores were extracted from each tumor, paired with NT tissue and mounted on a new recipient block using a semi-automated arraying device (TMArrayer, Pathology Devices, Westminster, MD, USA). IHC was performed on the TMA using a two-step immunoperoxidase technique. A TMED3 polyclonal antibody (Abcam, CA, USA) diluted 1:60 was used as the primary antibody. Briefly, after heating the sections in 10 mmol/l citrate buffer for antigen retrieval, the sections were incubated first with the primary antibody and then with the secondary antibody for one hour at room temperature. Finally, the sections were developed in diaminobenzidine solution under a microscope and counter-stained with hematoxylin. The IHC stains were assessed by three separate observers who had no knowledge of patient characteristics. TMED3 staining was abundant in the cytoplasm and nucleus. The expression was recorded after evaluating the staining intensity of positive cells (0 = none; 1 = + weak; 2 = ++ intermediate; 3 = +++ strong; 4 = ++++ very strong), as shown in Fig. S1A.

### RNA extraction, cDNA preparation and quantitative real-time PCR (qRT-PCR)

Total RNA was extracted from snap-frozen tissues using TRIzol reagent (Takara, Dalian, China) according to the manufacturer’s instructions. The quality of the total RNA was assessed using a Nanodrop 2000 and via agarose gel electrophoresis. First-strand cDNA was synthesized from 1–2 μg of total RNA using random primers and M-MLV Reverse Transcriptase (Invitrogen, CA). Real-time PCR was performed according to the SYBR Green protocol in a Step One Plus System (Applied Biosystems, Foster City, CA) with β-actin as the endogenous control. The TMED3-specific primers were 5′-GGGTTCTGTACCTGAGGAAA-3′ (forward) and 5′-CACCGAGGGTGAGCAGAT-3′ (reverse), and the β-actin-specific primers were 5′-TGACGTGGACATCCGCAAAG-3′ (forward) and 5′-CTGGAAGGTGGACAGCGAGG-3′ (reverse). The relative mRNA levels were calculated based on the Ct values and were normalized to β-actin expression.

### Cell culture

Human HCC cell lines were purchased from the Shanghai Institute of Life Sciences Cell Resource Center (Shanghai, China). All cell lines were cultured in Dulbecco’s Modified Eagle Medium (DMEM, HyClone, CA, USA) supplemented with 10% fetal bovine serum (FBS) and 1% penicillin/streptomycin (Gibco, CA, USA). All cell cultures were maintained at 37 °C in a humidified atmosphere with 5% CO_2_.

### Wound healing assays

For the wound healing assays, monolayer cells plated in 12-well plates were wounded with a plastic 200-μl pipette tip and then rinsed several times with medium to remove floating cells. The wound healing process was monitored using an inverted light microscope (Olympus).

### Western blot analysis

Total cell and tissue lysates were prepared in 1× sodium dodecyl sulfate buffer. Identical quantities of proteins were separated by sodium dodecyl sulfate-polyacrylamide gel electrophoresis and transferred onto nitrocellulose filter membranes. After incubation with antibodies specific for TMED3 (ab151056, ab173112; Abcam, CA, USA), IL-11 (ab187167; Abcam, CA, USA), STAT3 (ab119352; Abcam, CA, USA), P-STAT3 (ab76315; Abcam, CA, USA) or β-actin (ab8226; Abcam, CA, USA), the blots were incubated with IRDye 800-conjugated goat anti-rabbit IgG and IRDye 700-conjugated goat anti-mouse IgG, and the bands were detected using an Odyssey infrared scanner (Li-Cor). β-Actin was used as a loading control.

### Wound healing migration assay

Briefly, 1 × 10^5^ cells/well were plated in 6-well plates. After the cells attached, a wound was created in the middle of each well, and the medium was replaced with serum-free medium. The area of healing across the lesion was measured after a 48-h incubation.

### Transwell assays

Millicell 24-well culture insert plates (Millipore, USA) and polycarbonate membranes with a pore size of 8 μm were used for transwell assays. First, the insert plates were equilibrated with 0.5 ml of DMEM for 1 h at 37 °C in 5% CO_2_; then, the medium in the lower chambers was replaced with 0.5 ml of DMEM supplemented with 10% FBS. In total, 50,000 cells in 400 μl of serum-free DMEM were loaded into the upper chambers. After a 24-h incubation, the inserts were rinsed with PBS, and the upper surfaces of the membranes were scraped to remove the cells. The cells on the underside of the membrane were stained with Giemsa stain and counted under a microscope. Cells from each culture condition were examined in quadruplicate.

### Animal studies

To explore the effects of TEMD3 on tumor growth *in vivo*, 1 × 10^7^ HepG2 cells thatstably knocked down TMED3 and control cells were subcutaneouslyimplanted into the bilateral armpit of nine BALB/Cnude mice. The tumor volumes were measured everyweek after implantation (volume V = length × width ×length ×1/2). All mice were sacrificed five weeks later. A tailvein injection model was also used to evaluate the potentialof the cells to metastasize to the lungs. The metastaseswere monitored using an IVIS@ Lumina II system (CaliperLife Sciences, Hopkinton, MA) for 10 min after intraperitonealinjection of 4.0 mg of luciferin (Gold Biotech) in 50 μl of saline. Animals were housed in cages under standard conditions, and the methods were carried out following the requirements of the Second Military Medical University Animal Care Facility and the National Institutes of Health guidelines, and all experimental protocols were approved by the Institutional Animal Care and Use Committee of the Second Military Medical University, Shanghai, China. The mice were kept in pathogen-free conditions.

### Gene expression profiling and analysis

Total RNA was extracted using TRIzol Reagent (Life Technologies, Carlsbad, CA, US) according to the manufacturer’s instructions, and the RNA integrity number (RIN) was determined using an Agilent Bioanalyzer 2100 (Agilent Technologies, Santa Clara, CA, US). Acceptable total RNA preparations were further purified using an RNeasy Mini Kit (QIAGEN, GmBH, Germany) and RNase-Free DNase (QIAGEN, GmBH, Germany). Total RNA was amplified and labeled using a Low Input Quick Amp Labeling Kit, One-Color (Agilent Technologies, Santa Clara, CA, US) according to the manufacturer’s instructions. Labeled cRNA was purified using an RNeasy Mini Kit (QIAGEN, GmBH, Germany). Each slide was hybridized with 1.65 μg of Cy3-labeled cRNA in a hybridization oven using the Gene Expression Hybridization Kit (Agilent Technologies, Santa Clara, CA, US) according to the manufacturer’s instructions. After 17 h of hybridization, slides were washed in staining dishes (Thermo Shandon, Waltham, MA, US) using the Gene Expression Wash Buffer Kit (Agilent Technologies, Santa Clara, CA, US) according to the manufacturer’s instructions. Slides were scanned using an Agilent Microarray Scanner (Agilent Technologies, Santa Clara, CA, US) with the default settings. Data were extracted with Feature Extraction Software 10.7 (Agilent Technologies, Santa Clara, CA, US) at the Biotechnology Corporation, Shanghai, PR China. Raw data were normalized using the Quantile algorithm in GeneSpring 11.0 (Agilent Technologies, Santa Clara, CA, US), and we considered genes that were up- or down-regulated by more than 1.5-fold in two independent biological duplicates.

### IL-11 ELISA

IL-11 levels in normal culture medium collected after 48 h from different cells were detected with the Human IL-11 ELISA Kit (ab189569; Abcam, CA, USA) according to the manufacturer’s instructions.

### Statistical analysis

Statistical analyses were performed using SPSS 18.0 and GraphPad Prism 5.0. Numerical data are presented as the mean and standard error. Differences between proportions were evaluated using the paired Student’s t-test. The correlations between TMED3 expression and clinicopathologic parameters were evaluated using chi-square tests, and survival analyses were evaluated by Kaplan–Meier analysis. P values ≤ 0.05 were considered statistically significant. Each experiment was repeated at least three times.

### Data and materials availability

The microarray datasets have been deposited in the Gene Expression Omnibus (GEO) under GSE88772 (https://www.ncbi.nlm.nih.gov/geo/query/acc.cgi?acc=GSE88772).

## Additional Information

**How to cite this article**: Zheng, H. *et al*. TMED3 promotes hepatocellular carcinoma progression via IL-11/STAT3 signaling. *Sci. Rep.* **6**, 37070; doi: 10.1038/srep37070 (2016).

**Publisher's note:** Springer Nature remains neutral with regard to jurisdictional claims in published maps and institutional affiliations.

## Supplementary Material

Supplementary Information

## Figures and Tables

**Table 1:** Clinical characteristics of 313 HCC patients according to TMED3 expression level.

	TMED3^a^		χ^2^	P value
	High	Low		
All cases				
Age, y			0.001	0.981
≥55	70	47		
<55	117	79		
Gender			0.340	0.854
Male	166	111		
Female	21	15		
HBsAg			2.358	0.125
Positive	167	105		
Negative	20	21		
HBeAg			0.527	0.468
Positive	42	24		
Negative	145	102		
AFP, μg/L			5.384	0.020
≥20	128	70		
<20	59	56		
Tumor size, cm			4.013	0.045
≥5	112	61		
<5	75	65		
Tumor number			0.011	0.917
Single	149	101		
Multiple	38	25		
Vascular invasion			62.822	<0.001
Present	146	42		
Absent	41	84		
Tumor differentiation			2.393	0.122
I–II	16	18		
III–IV	168	108		

Values in red are statistically significant (P < 0.05).

AFP, alpha-fetoprotein; HBsAg, hepatitis B surface antigen; HBeAg, hepatitis B e antigen.

^a^Patients whose tumor tissue immunohistochemistry score was >2 were included in the high expression group, and the remaining patients comprised the low expression group.

**Table 2:** Univariate analysis of the risk factors for HCC recurrence and overall survival.

Variate	Recurrence Free Survival		Overall Survival	
	Hazard ratio	P value	Hazard ratio	P value
TMED3 (High expression)	1.520 (1.127–2.049)	0.006	1.547 (1.099–2.178)	0.012
Tumor size (>5 cm)	1.449 (1.081–1.941)	0.013	1.861 (1.323–2.616)	<0.001
Tumor number (Multiple)			1.583 (1.090–2.300)	0.016
AFP (positive)	1.503 (1.107–2.042)	0.009	1.453 (1.026–2.056)	0.035
HBeAg (positive)	2.012 (1.204–3.361)	0.008	2.130 (1.152–3.940)	0.016
HBsAg (positive)	1.548 (1.116–2.146)	0.009	1.499 (1.042–2.158)	0.029
Vascular invasion	1.580 (1.168–2.136)	0.003	1.547(1.116–2.220)	0.010

**Table 3:** Multivariate analysis of risk factors for HCC recurrence and overall survival.

Variable	Recurrence-free Survival		Overall Survival	
	Hazard ratio	P value	Hazard ratio	P value
TMED3 (high expression)	1.403 (1.038–1.898)	0.028	1.446 (1.025–2.040)	0.035
Tumor size (>5 cm)	1.503 (1.118–2.020)	0.007	1.841 (1.306–2.594)	<0.001
Tumor number (Multiple)			1.587 (1.091–2.308)	0.016
HBeAg (positive)	1.523 (1.095–2.118)	0.012		
HBsAg (positive)	2.016 (1.204–3.374)	0.008	2.322 (1.251–4.310)	0.008
