# Supplementary material for: TMED3 promotes hepatocellular carcinoma progression via IL-11/STAT3 signaling
Source: Sci Rep. 2016 Nov 30;6:37070. doi: 10.1038/srep37070 (PMC5128793; doi:10.1038/srep37070)
Supplement: Supplementary Information [file srep37070-s1.doc]

**Supplemental Data**

**TMED3 promotes hepatocellular carcinoma progression via IL-11/STAT3 signaling**

Hao Zheng^1#^, Yuan Yang^1,2#^, Jun Han^1,3#^, Wei-hua Jiang^4^, Cheng Chen^5^, Meng-chao Wang^1^, Rong Gao^3^, Shuai Li^6^, Tao Tian^1^, Jian Wang^1^, Li-jun Ma^4^*, Hao Ren^3^* and Wei‑ping Zhou^1^*

**Figure legends**

**FigureS1.TMED3 expression is up-regulated in HCC samples**

A. Representative IHC images for each score: representative IHC images with scores of 0, 1, 2, 3, and 4, respectively; 4, 3, and 2 show tumor tissues, and 1 and 0 showed jacent noncancerous tissue.

B. Representative images of IHC staining for TMED3.

C. Distribution of IHC scores for tumor and paired noncancerous tissues.

**FigureS2.Stable knockdown and overexpression of TMED3 inHepG2and Huh7 cells**

A.TMED3 expression levels in various liver and HCC cell lines were determined by qRT-PCR and normalized to β-actin expression.

B and C. qRT-PCR was performed to assess the stable knockdown and overexpression of TMED3 in HepG2 and Huh7 cells, respectively, which were established using a lentiviral system.

**Figure S3.TMED3 did not promote cell proliferation in vivo**

Xenografts at 5 weeks after SC transplantation ofsi-TMED3 or control HepG2cells (n=5): A, images; B, tumor growth.

**Figure S4. Representative IHC staining of TMED3 and P-STAT3 in serial sections. Note the positive correlation between TMED3 and P-STAT3 levels in the clinical samples**

**
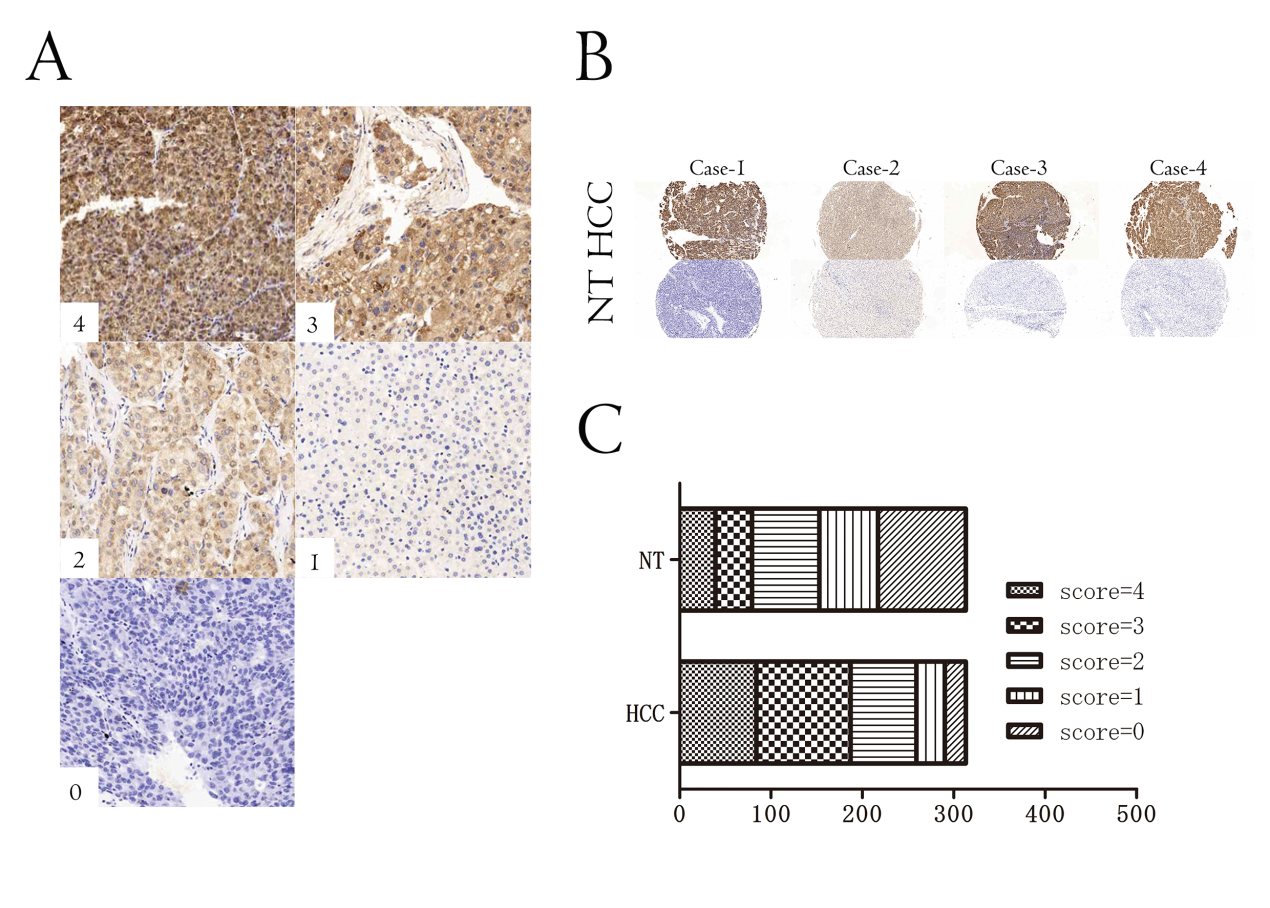
**

Supplement-figure1


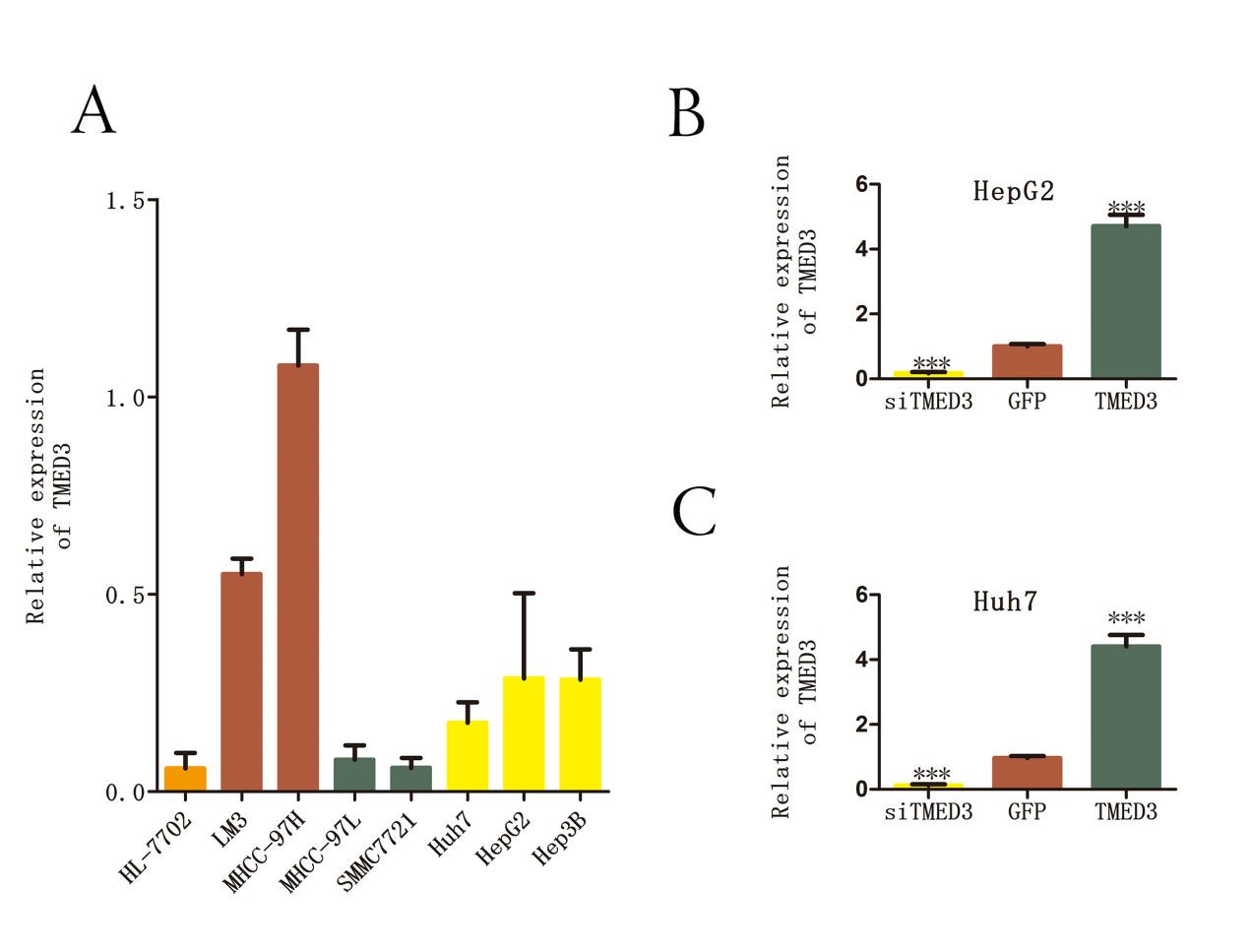


Supplement-figure2


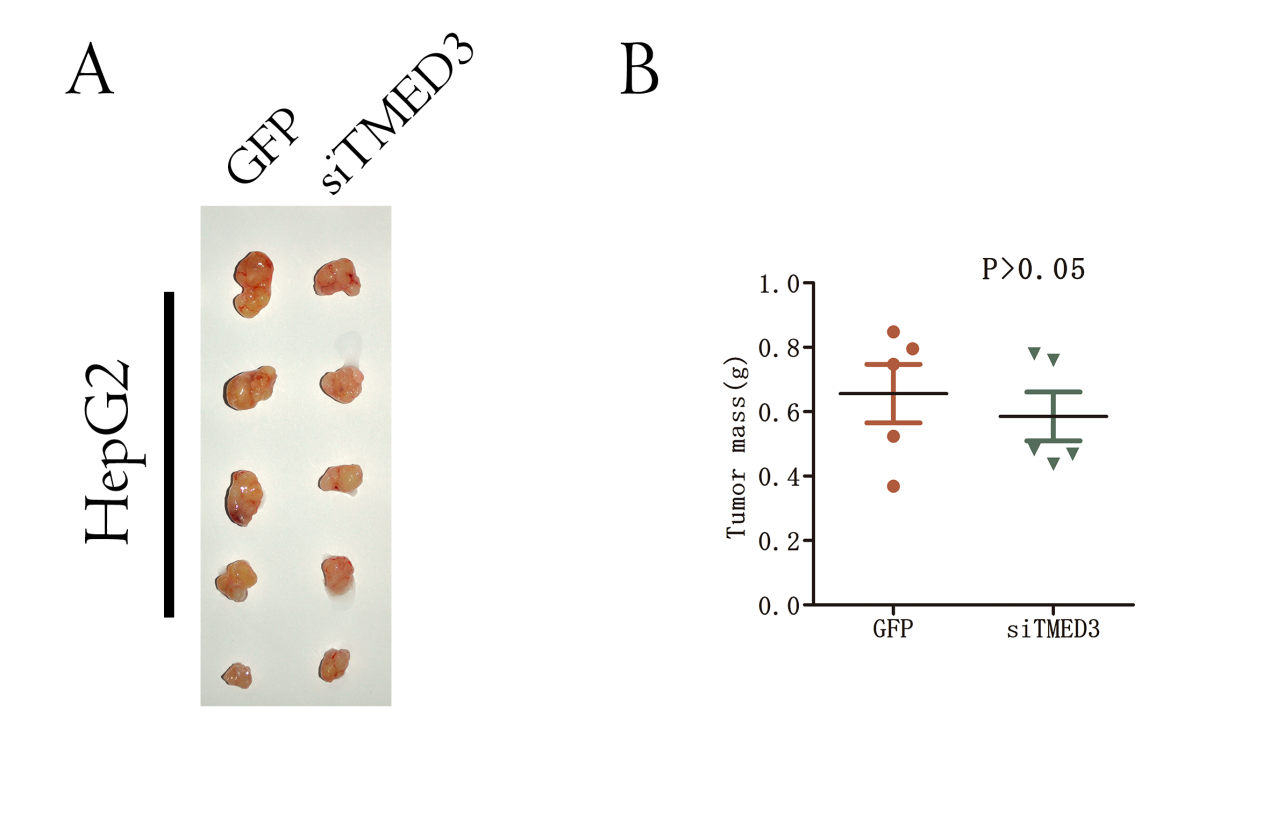


Supplement-figure3


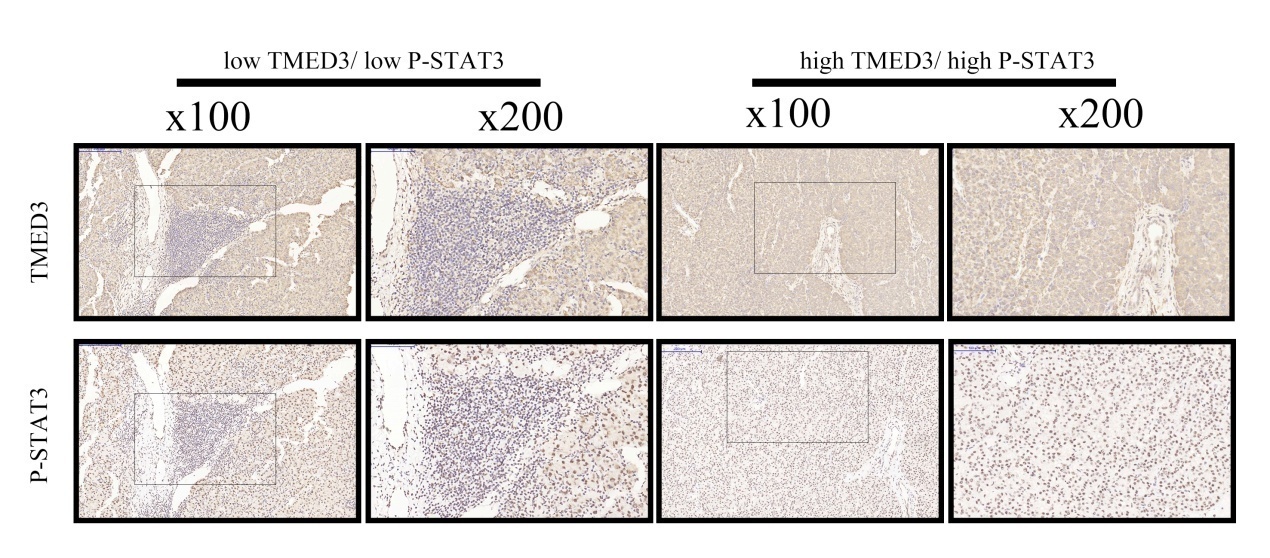


Supplement-figure4

**Supplement-Table1**

**Summary of 60 HCC patients Clinical Clinicopathologic** Characteristics

| Characteristics | No.of patients |
| --- | --- |
| All cases |  |
| Age, y |  |
| ≥ 55 | 45 |
| < 55 | 15 |
| Gender |  |
| Male | 50 |
| Female | 10 |
| HBsAg |  |
| Positive | 55 |
| Negative | 5 |
| HBeAg |  |
| Positive | 21 |
| Negative | 39 |
| AFP, μg/L |  |
| ≥ 20 | 46 |
| < 20 | 14 |
| Tumor size, cm |  |
| ≥ 5 | 19 |
| < 5 | 41 |
| Tumor number |  |
| Single | 48 |
| Multiple | 12 |
| Vascular invasion |  |
| Present | 23 |
| Absent | 37 |
| Tumor differentiation |  |
| I-II | 22 |
| III-IV | 38 |

AFP, alpha-fetoprotein; HBsAg, hepatitis B surface antigen; HBeAg, hepatitis B e antigen.

**Supplement-Table2**

**Summary of 30 HCC patients Clinical Clinicopathologic** Characteristics

| Characteristics | No.of patients |
| --- | --- |
| All cases |  |
| Age, y |  |
| ≥ 55 | 26 |
| < 55 | 4 |
| Gender |  |
| Male | 25 |
| Female | 5 |
| HBsAg |  |
| Positive | 21 |
| Negative | 9 |
| HBeAg |  |
| Positive | 20 |
| Negative | 10 |
| AFP, μg/L |  |
| ≥ 20 | 26 |
| < 20 | 4 |
| Tumor size, cm |  |
| ≥ 5 | 15 |
| < 5 | 15 |
| Tumor number |  |
| Single | 22 |
| Multiple | 8 |
| Vascular invasion |  |
| Present | 30 |
| Absent | 0 |
| Tumor differentiation |  |
| I-II | 19 |
| III-IV | 11 |

AFP, alpha-fetoprotein; HBsAg, hepatitis B surface antigen; HBeAg, hepatitis B e antigen.
